# Psychological distress as predictor of quality of life in men experiencing infertility: a cross-sectional survey

**DOI:** 10.1186/1742-4755-7-3

**Published:** 2010-05-09

**Authors:** Juliana LR Chachamovich, Eduardo Chachamovich, Hélène Ezer, Fernanda P Cordova, Marcelo MP Fleck, Daniela R Knauth, Eduardo P Passos

**Affiliations:** 1 School of Nursing, McGill University, Wilson Hall 3506 University Street, Montreal, QC, H3A 2A7, Canadá; 2 McGill Group for Suicide Studies, Douglas Mental Health Institute, McGill University, 6875 LaSalle blvd., Montreal, QC, H4H 1R3, Canada; 3 School of Nursing, Federal University of Rio Grande do Sul, Rua São Manoel, 963, Porto Alegre, RS, 90620-110, Brazil; 4 Post-Graduate Program in Psychiatry, Federal University of Rio Grande do Sul, Brazil. Rua Ramiro Barcelos, 2400, 2 andar. Porto Alegre, RS, CEP 90300-050, Brazil; 5 Social Medicine Department, Federal University of Rio Grande do Sul, Brazil. Rua Ramiro Barcelos, 2400, 2 andar. Porto Alegre, RS, CEP 90300-050, Brazil; 6 Post-Graduate Program in Medicine, Assisted Reproduction Service, Gynecology and Obstetrics Department. Rua Ramiro Barcelos 2400, Porto Alegre, RS, CEP 90300-050, Brazil

## Abstract

**Background:**

Infertility is associated with impairment in human life. The quality of life (QOL) construct allows measuring the impact of health conditions in a broader way. The study aimed to explore the impact of the psychological distress on QOL's dimensions in men experiencing infertility.

**Methods:**

162 men were completed a socio-demographic form, SF-36, WHOQOL-BREF, Beck Anxiety Inventory and Beck Depression Inventory. Hierarchical regressions included demographic and clinic variables, and subsequently depression and anxiety were added.

**Results and Discussion:**

Model 1 was not accurate in predicting QOL. R^2 ^values ranged from 0.029 (Social Functioning) to 0.149 (Mental Health). Eight domains were not associated with any of the predictors. In the second model, a R^2^increase was observed in all domains. R^2 ^of QOL scores ranged from .209 (Role Physical) to .406 (Social Functioning). The intensity of the depression was a significant predictor for all outcomes. The load of depression was higher than the ones of the socio-demographic and clinical variables. Anxiety levels have also presented the same effect, but with less intensity.

**Conclusion:**

Subthreshold depression and anxiety were major predictors of QOL in men experiencing infertility. Health professionals need to include assessment of psychological symptomatology to plan more efficient interventions to infertile patients.

## Background

Investigations on infertility are voluminous, and have shown that involuntary childlessness can be devastating, and it is associated with psychological distress[1]. The effects of infertility seem to be comprehensive, and are not restricted to sexual or reproductive areas of life[2], but its impact burden on several psychosocial areas of human existence [1,3-6]. Impairments have been reported regarding distinct aspects, such as psychopathology [7], relationship abilities [8], marital life [9,10], family life [11], and economic terms [8].

There is an increasing interest in exploring infertility in a comprehensive way, taking into account the plethora of associated subjective perceptions. A systematic approach is needed to measure this phenomenon and allow comparability of studies. Quality of life (QOL) has emerged as a well-established concept to address these issues. Being considered a restatement of the World Health Organization's commitment to the promotion of a holistic approach to health and healthcare [12], QOL assumes a particular relevance when clinicians and researchers intend to investigate complex and multidimensional health conditions [2]. QOL assessments include aspects of health status, psychological well-being, physical and social functioning, and environmental and spiritual facets [13-16].

While the findings on QOL among infertile women have shown mainly agreement, this seems not to be the case among men. Studies on men's QOL have demonstrated inconclusive findings. Comparisons between men of infertile couples and normative data [17] showed no differences between these groups in Italy, whereas lower Mental, Emotional and Social scores were found in the US[6] and Netherlands[18], respectively. Studies on QOL's predictors in men have described that educational level, age, marital relationship, previous In Vitro Fertilization attempts, and duration of infertility are associated with lower scores in Mental and Emotional domains [18-20].

It has been demonstrated that QOL is particularly vulnerable to depressive and anxiety symptoms [21-23]. Not only a full-blown depressive diagnosis has a determinant impact on all QOL domains, but also subsyndromal symptoms may affect QOL [24,25]. Since infertile patients are at higher risk to have depressive and anxious symptoms[7], we hypothesize that discrepant findings may be related to different levels of anxiety and depressive symptoms across the studies' samples. Thus, depression and anxiety might mediate the relationship of the tested predictors and the QOL outcomes. The present study aimed to explore the potential impact of anxiety and depression on the QOL of men experiencing infertility. In addition, we examined which predictors of QOL remain relevant if anxiety and depression symptoms are controlled for.

## Methods

### Subjects

163 male patients seen at the assisted reproduction service of a university hospital were asked to take part in this cross-sectional study. The subjects were interviewed while waiting for medical visits. Patients were enrolled if they were seeking investigation for infertility, and if they had been unable to conceive after at least one year of unprotected sexual intercourses. Subjects with scores above the clinical cut-point were excluded from the analyses. All respondents were informed about the objectives of the study and the confidentiality of the data. The project was approved by the research ethics committee of the university hospital, which follows the Helsinki declaration, as revised in 1983. One subject declined to participate in the study. The final sample came to 162 men.

### Procedures

The following instruments carried out face-to-face interviews:

1) A socio-demographic and clinical data form, which assesses marital status, length of relationship with the present partner, changes in dialogue with the partner, socio-economic status (assessed by a standardized rating scale issued by the Brazilian Association of Market Research Institutes), age, educational level, perceived etiology of infertility, medical diagnosis of the etiology of infertility, duration of conception attempts, number of previous attempts at reproduction techniques, type of assisted reproduction technique and sexual life satisfaction (self-reported);

2) The WHOQOL-BREF, which is a generic QOL assessment instrument, developed by the WHO [26]. It has been translated and validated into Portuguese [27] and provides an overall score for QOL, as well as individual scores by domain. Its four domains are physical health, psychological health, social relationships and environment. A large number of studies have proved its suitability to assess QOL in several health conditions, including infertility [2].

3) The Health Survey Short Form (SF-36), which is a multidimensional questionnaire of QOL. SF-36 has been used in studies in different areas, including infertility [2,6,17,19,20,28]. It is comprised of eight domains (physical functioning, role physical, social functioning, bodily pain, mental health, role emotional, vitality and general health) [29]. It has been validated into Portuguese [30].

4) Beck Depression Inventory (BDI): is an instrument that measures intensity of depression [31]. It has also been validated in Portuguese [32]. The total BDI score is obtained from the sum of 21 items that assess both the Cognitive-Affective and the Somatic-Performance aspects of depression. Scores under 19 represent absence of major depression; from 19 to 29 moderate depression and above 30, severe depression [33].

5) Beck Anxiety Inventory (BAI): is a 21-item Likert self-report questionnaire measuring common symptoms of clinical anxiety. Thirteen items assess physiological symptoms, five describe cognitive aspects, and three represent both somatic and cognitive symptoms. Scores above 10 suggest mild anxiety, with 19 reflecting moderate anxiety, and 30 indicating severe anxiety. The validated Portuguese version was used [32].

### Statistical Analysis

Skewness and kurtosis of the QOL scores were checked to detect important departures from normality. Values between -2 and +2 indicated that no severe departure from normality. This finding was corroborated by the analysis of the normal P-P and Q-Q plots.

Hierarchical linear multiple regressions were applied in each domain of WHOQOL-BREF and SF-36 instruments to detect the impact of depression and anxiety. The first model included age, educational level, marital relationship, duration of the marital relationship, duration of attempts to conceive, sexual life, perceived etiology of infertility, previous assisted reproduction technique, and having a child (or not) as independent variables. The second model added depression and anxiety levels as independent levels too.

A conservative approach was assumed to include independent variables in the multivariate analyses. We have opted not to exclude the variables that fail to show significant results in univariate analyses, since high *p *values at this stage do not necessarily mean that these variables would not be relevant for the multivariate model. In opposite, we chose to include them in the multivariate analyses and test whether they prove to be relevant or not. In addition, most of them have been indicated as significant in other publications [18,19].

Variance Inflation Factor (VIF) was inspected for multicolinearity in each model, with results higher than 10 being considered as indicative of this problem[34]. Results were described through R^2 ^changes and standardized β-values. Significance was set at an alpha level of 0.05.

Sample size was estimated based on the directions by Norman and Streiner [35]. For multiple linear regressions, the sample should include a minimum of 10 subjects per each independent variable. Since the tested multivariate model comprised 12 predictors, a minimum of 120 subjects was required.

## Results

### Demographics

Regarding the sexual life satisfaction, the majority of subjects reported no alterations (74.7%), while 21.6% considered that the sexual life was better, and 3.7% indicated dissatisfaction. For 69.8% of the sample, the dialogue with the partner remained constant; 29.0% reported an increase of the quality of the dialogue, and only 1.2% declared that the dialogue had become worse after infertility was noticed. Table 1 describes the characteristics of the sample.

**Table 1:** Total sample characteristics (n = 162)

Demographics	N (%) OR Mean (SD)
**Age (years)**	36.1 (7.69)
**Marital Status**	
Legally married	85 (53.5)
Living with partner	74 (46.5)
	
**Duration of relationship (years)**	9.13 (4.72)
**Educational Level**	
< 9 years	47 (29)
9-11 years	68 (42)
> 11 years	47 (29)
**Perceived etiology of infertility**	
Male	47 (29)
Female	65 (40.1)
Both	23 (14.2)
Unknown	27 (16.6)
**Previous Assisted Reproduction**	
No	136 (84)
One time	14 (8.6)
More than one	12 (7.4)
**Type of Assisted Reproduction**	
Artificial Insemination	17 (10.5)
In Vitro Fertilization	13 (8)
Intra cytoplasmic sperm injection	6 (3.7)
Ovulation Induction	1 (0.6)
**Having at least one child**	
No	124 (76.5)
Yes	38 (23.5)
**Duration of attempt to conceive**	
	
< 2 years	28 (17.7)
2-5 years	56 (35.4)
> 5 years	74 (46.8)
**Socio-Economic Status**	
Class A	11 (6.8)
Class B	88 (54.3)
Class C	61 (37.7)
Class D	2 (1.2)
**BDI**	4.74 (5.19)
**BAI**	5.70 (5.81)
**QOL Domains**	
	
Physical Domain	78.39 (12.31)
	
Psychological Domain	74.71 (12.10)
	
Environment Domain	61.70 (13.56)
	
Social Relations Domain	72.50 (16.04)
	
Overall Score	73.99 (14.34)

BDI = Beck Depression Inventory, BAI = Beck Anxiety Inventory, QOL = Quality of Life

The low BDI and BAI mean scores indicate a predominately non-depressed and non-anxious sample. Depression and anxiety levels were subclinical in 98.1% and 96.3% of the subjects, respectively. Only 1.9% and 3.7% of the sample presented scores above the cut-point for depression and anxiety, respectively. No severe cases of depression or anxiety were observed. The quality of life scores varied from 61.7 (SD 13.56) to 78.39 (SD 12.31), indicating moderate to high levels.

### Multivariate Analyses

The clinical and socio-demographical variables were included in the multivariate analyses as predictors, and the QOL scores of each domain were assigned as outcomes. All QOL scores presented normal distribution and no multicolinearity (VIF equal or lower than 1.6).

The etiology of the infertility was investigated by two means. First, men were required to report the subjective perception of the etiology (i.e., whether they believed that the etiology was feminine, masculine, both or unknown). Secondly, the medical chart of each subject was revised for the medical diagnosis of infertility. No differences were found between the medical diagnosis and the patient's perception of the etiology (χ^2 ^= 0.705, p = 0.872, df = 3). Thus, the subjective perception of the etiology was kept for the multivariate stage. In addition to the statistical similarity between them, the subjective perception is more likely to have an effective impact of patient's quality of life (rather than a established diagnosis that the patient could not be aware of). For the multivariate analysis, etiology was entered as either a male or non-male factor.

The model 1 tested the load of each socio-demographic variable to predict QOL in thirteen scores (5 from WHOQOL-BREF and 8 from SF-36). Independent variables were selected because they represent important demographic variables and are widely reported in several studies [17-19,28]. Models' coefficient of determination and standardized-β coefficients are described in Table 2. A close inspection on the results revealed that this model proved not to be accurate in predicting quality of life. Coefficients of determination were low. R^2 ^values ranged from 0.029 (Social Functioning Domain) to 0.149 (Mental Health Domain). Moreover, eight out of the 13 domains scores were not predicted by any of the independent variables. Among the independent variables, socio-economic status and changes in dialogue with partner were the most relevant ones, predicting the scores of two domains each (Environmental and General Health; and Environmental and Mental Health, respectively).

**Table 2:** R^2 ^**values and standardized β-coefficients in multiple linear regressions for each WHOQOL-BREF and SF36 domains (model 1)**

	WHOQOL-BREF					SF-36							
	Psychol Std-β	Physical Std-β	Social Std-β	Environ Std-β	Overall Std-β	Phys Fun Std-β	R Phys Std-β	B Pain Std-β	G Health Std-β	Vitality Std-β	S Funct Std-β	R Emot Std-β	Ment H Std-β
Age	.037	-.059	.056	-.010	.055	**-.204**	-.118	-.046	.023	-.083	-.005	.079	.143
Duration of the relationship	.042	.104	.037	.117	.014	.049	.055	.023	.042	.180	.042	.021	**.203**
Educational level	.038	-.087	-.125	-.064	-.127	.020	.102	-.079	.032	-.082	-.080	**-.203**	-.172
Sexual life	.072	.134	.000	-.015	.108	.080	.071	.096	.010	.095	.027	.018	-.032
SES	-.095	-.185	-.158	**-.196**	-.148	.048	-.004	-.146	**-.249**	.038	-.128	-.002	-.035
Having children	.061	.011	.117	-.005	-.061	-.036	-.018	.088	-.017	.095	.043	.020	.014
Previous AR	-.004	-.049	.045	**.249**	.093	.097	-.011	.104	.049	.009	.066	.094	.144
Dialogue with partner	.173	-.018	.118	**.181**	.106	.031	-.135	.069	.116	.161	.051	.010	**.273**
Duration of attempts	-.054	-.041	.045	-.162	-.011	.00	-.038	-.122	-.106	.065	-.038	.002	-.054
Etiology	-.022	-.002	-.119	-.057	-.065	.158	-.039	-.079	.003	.064	-.052	-.044	-.038

Model R^2^	.070	.059	.081	.145	.067	.090	.040	.073	.107	.107	.029	.044	.149

Psychol = Psychological; Environ = Environmental; Phys Fun = Physical Functioning; R Phys = Role Physical; B Pain = Bodily Pain; S Funct = Social Functioning; R Emot = Role Emotional; Ment H = Mental Health; educational level (1 = < 9 years; 2 = 9-11 years, 3 = > 11 years); sexual life (1 = worse, 2 = equal, 3 = better); SES = socio-economic status (1 = A, 2 = B, 3 = C, 4 = D); Having children (1 = no; 2 = yes); Previous AR = Previous Assisted Reproduction (1 = no, 2 = yes); dialogue with partner(1 = worse, 2 = equal, 3 = better); etiology (1 = male, 2 = non-male); BAI = Beck Anxiety Inventory; BDI = Beck Depression Inventory.

Bolded values are significant (p < .05)

Subsequently, the model 2 tested the effect of the inclusion of depressive and anxiety symptoms in the multivariate model. Table 3 illustrates the results of the model 2.

**Table 3:** R^2 ^values and standardized β-coefficients in multiple linear regressions for each WHOQOL-BREF and SF36 domains (model 2)

	WHOQOL-BREF					SF-36							
	Psychol Std-β	Physical Std-β	Social Std-β	Environ Std-β	Overall Std-β	Phys Fun Std-β	R Phys Std-β	B Pain Std-β	G Health Std-β	Vitality Std-β	S Funct Std-β	R Emot Std-β	Ment H Std-β
Age	.084	.000	.095	.039	.100	-.154	-.075	.013	.063	.003	.072	.122	**.201**
Duration of the relationship	.010	.069	.003	.089	-.018	.024	.019	-.014	.016	.134	.010	-.018	**.166**
Educational level	.063	-.052	-.113	-.033	-.105	.054	.117	-.010	.053	-.040	-.019	**-.190**	-.139
Sexual life	.058	.115	-.009	-.032	.095	.062	.061	.071	-.002	.073	-.004	.009	.051
SES	-.024	-.097	-.099	-.124	-.080	.121	.062	-.037	**-.189**	.121	-.017	.065	.051
Having Children	.015	-.045	.076	-.050	-.105	-.080	-.062	.032	-.055	.030	-.023	-.025	-.041
Previous AR	-.014	-.069	.047	**.230**	.085	.074	-.011	.064	.038	-.013	.019	.097	.128
Dialogue with partner	.109	-.097	.062	.116	.044	-.034	**-.196**	-.020	.062	.090	-.047	-.052	**.194**
Duration of attempts	-.100	-.097	.005	**-.208**	-.056	.015	-.082	**-.172**	-.145	.015	-.107	-.043	-.110
Etiology of infertility	-.019	-.001	-.112	-.057	-.062	**.155**	-.032	-.080	.005	.069	-.060	-.035	-.036

**BAI total**	**-.199**	**-.280**	-.108	**-.242**	-.180	**-.269**	-.131	**-.418**	-.176	**-.293**	**-.472**	-.115	**-.261**
**BDI total**	**-.294**	**-.314**	**-.323**	**-.246**	**-.293**	**-.213**	**-.340**	**-.252**	**-.233**	**-.239**	**-.249**	**-.369**	**-.333**

Model R^2^	.246	.311	.225	.315	.230	.255	.209	.397	.228	.304	.406	.226	.403
R^2 ^Change (Model 2 - Model 1)	.176	.252	.144	.170	.163	.165	.169	.324	.121	.197	.377	.182	.254

Psychol = Psychological; Environ = Environmental; Phys Fun = Physical Functioning; R Phys = Role Physical; B Pain = Bodily Pain; S Funct = Social Functioning; R Emot = Role Emotional; Ment H = Mental Health; educational level (1 = < 9 years; 2 = 9-11 years, 3 = > 11 years); sexual life (1 = worse, 2 = equal, 3 = better); SES = socio-economic status (1 = A, 2 = B, 3 = C, 4 = D); Having children (1 = no; 2 = yes); Previous AR = Previous Assisted Reproduction (1 = no, 2 = yes); dialogue with partner(1 = worse, 2 = equal, 3 = better); etiology (1 = male, 2 = non-male); BAI = Beck Anxiety Inventory; BDI = Beck Depression Inventory.

Bolded values are significant (p < .05)

A consistent effect was observed. The coefficient of determination increased in all domains. The percentage of the explained variance of the QOL scores ranged from .209 (Role Physical Domain) to .406 (Social Functioning Domain). The intensity of the depression symptoms proved to be a significant predictor for all the 13 outcomes. The load of depression symptoms was substantially higher than the ones of the socio-demographic and clinical variables, as shown by the standardized β-coefficients. The anxiety levels have also presented the same effect, but with less intensity. They were significant predictors in 8 out of 13 domains, and their standardized β-coefficients were consistently lower than the BDI ones. In addition, the inclusion of BDI and BAI in the multivariate model led to changes in the significance of some socio-demographic and clinical variables. For example, age was not a predictor of Mental Health in model 1, but proved to be a significant variable when anxiety and depression were included in the analysis. In opposite, it was indicated as a significant variable for Physical Functioning in the first model, but the inclusion of BDI and BAI resulted in a non-significant effect of age in the model 2 (i.e., age was not significant when depression and anxiety were controlled).

## Discussion

Our results suggest that even minimal levels of depression and anxiety are major predictors of QOL, and have a much higher load than socio-demographical and clinical variables. Some of these socio-demographic and clinical variables proved to be non-significant when the model is controlled for anxiety and depression suggesting that the associations between these are probably influenced by anxiety and depression.

The majority of the subjects presented very low levels of depression and anxiety. Although somewhat surprising, this finding could be related to the obstructed access to assisted reproductive treatments in Brazil. In Brazil, the treatment is freely offered by the public health system, but the medication has to be provided by the couples and is very expensive. There is a considerable delay in setting appointments and referrals to assisted reproduction clinics. As a consequence, patients that are able to pursuit the treatment and afford it are not representative of the infertile population, but rather have a higher socio-economic status and are less likely to be significantly depressed or anxious. We could then hypothesize that the obstructions to treatment can act as a filter to severe psychopathology conditions.

The explained variance of the QOL scores relied on the anxiety and depression levels. The coefficients of determination observed in the model 1 were modest. In our study, the socio-demographic and clinical variables were not able to explain more than 15% of the QOL variance. These variables have been used in other investigations, and proved to be significant predictors. For example, poor marital relationship, educational level, age and duration of attempts were described as predictors in studies using multivariate approaches [17-19]. However, these studies do not report to what extent the multivariate models are explained by these independent variables. Furthermore, among our findings Environment and Mental Health presented the highest R^2 ^values. Concomitantly, only few QOL domains were predicted by this set of independent variables.

Significant improvements were observed when depression and anxiety level were included in the multivariate model. R^2 ^increased markedly, and the Δ R^2 ^accounts for almost all the model 2 coefficient of determination. This ultimately emphasizes that the load of psychological distress is higher than the socio-demographic and clinical ones. This phenomenon has been consistently demonstrated in clinical and non-clinical samples, but has not been reported in infertile subjects up to the present. Our group has already [24] described that clinical and subclinical depressive symptoms are the most important predictors of QOL in a large international sample of older adults, and showed that the QOL models were significantly improved when depression was included. Similar findings were reported in a sample of patients seeking primary care units [25] and in a nationwide sample in Kuwait [22].

While the R^2 ^of the models without anxiety and depression were low, the ones from the model 2 were compatible to those reported in literature. QOL is a comprehensive construct, and requires complex models to provide accurate explanation [36]. Recent investigations that used multivariate approach on QOL reported model R^2 ^values of .568 [37], .475 [38] and .214-.476 [24]. The present R^2 ^values ranged from .209 (Role Physical) to .406 (Social Functioning). These values are considered sizeable given the complex nature of the outcomes [36].

Predictors were altered from the model 1 to 2. The inclusion of depression and anxiety in the multivariate models determined a distinct pattern of predictors. Noteworthy, the intensity of depression and anxiety in our sample is minimal. It is expected that the load of this symptomatology is consistently higher in subjects with moderate or severe depression and/or anxiety.

The last decade has witnessed a sustained increase of the published data on quality of life and infertility. The QOL impairment among infertile women has been extensively reported [2,18,19,28,39-43]. Infertility has a pervasive impact in women with involuntary childlessness [1,2,28,39,40]. This pattern reflects in decreased scores in all QOL domains [2,17-19,28]. Moreover, studies with couples have highlighted that the impact of infertility is more intense in women than men [17-20,28]. Although the reported findings on QOL in infertile women have shown agreement in different cultural settings, this seems not to be the case among men. The scarce data published tend to reveal discrepancies with different study designs. Comparisons of QOL between men of infertile couples and normative data were run in three countries. In Italy, Ragni et al. [17] showed no differences between these two groups, whereas statistically lower scores were found in the Netherlands [18] and the US [6]. The former reported impairment in Emotional Behavior and Social Functioning domains, and the latter demonstrated lower Mental Health scores. Moreover, few studies have also explored the predictors of QOL in infertile men. They have showed that educational level, age, marital relationship, previous In Vitro Fertilization attempts, and duration of infertility were associated with lower scores in Mental Health and Emotional Behavior domains [18-20]. Interestingly, the studies that were able to detect significant predictors revealed that only the mental and/or emotional aspects were affected in men.

Studies on infertility and psychosocial impact among men represent a recent field of interest, since by several reasons women had been firstly investigated. To examine the aspects that compose the infertile men's experience is the first step to establish health interventions. In the current study, psychological distress determined an effect on the model fit and on the previous predictors. Depression levels had a more consisted impact than anxiety. This pattern is corroborated by a recent study with a national sample in Kuwait. Authors indicated that major depressive disorder has a higher impact than generalized anxiety disorder in QOL, as measured by the WHOQOL-BREF [22]. These findings assume particular relevance because the instruments that were used are indeed accurate for assessing QOL, depression and anxiety, and their psychometric performance have been extensively reported. Furthermore, the QOL instruments are representative of two complementary theoretical conceptualizations (the functional model and the satisfaction model). This ensures the validity of the present findings.

Some demographic and clinical variables showed significant power of prediction in the final model. Age showed a positive association with Mental Health, suggesting that younger men are more likely to experience QOL impairments than older ones. This finding is in line to the one described by Fekkes et al. [18] among subjects planning IVF. This domain was also associated with length of the relationship, suggesting that longer relationships are linked to higher QOL. Educational level was negatively correlated to Role Emotional.

Previous assisted reproduction attempt was associated with Environment domain. This domain is closely related to concrete issues (rather than abstract satisfaction factors) [26,44], and is more likely to be affected by financial aspects. In developing countries, assisted reproduction often are only available to the ones with more financial resources [45]. As commented above, it is expected that subjects that are seeking for assisted reproduction in Brazil are somewhat wealthier than the general population, and more satisfied with the environmental issues included in this domain.

Changes in dialogue with partner was a significant predictor for Mental Health and Role Physical domains, being positively associated with the former and negatively associated with the latter. The impairment of Mental Health among men who report problems in dialoguing with partner was also described by Lau et al [19]. The duration of attempts was negatively associated with Environment and Bodily Pain domains. This variable has been cited as a predictor of low QOL among infertile women [17], but there were no data among men up to the present.

Men who perceived that the infertility had not a male-related etiology had a higher QOL score in the Physical Functioning domain. It suggests that when the subject believes that he is not the responsible for the inability to conceive, he reports a better QOL. Lau et al. reported that infertile women who attribute the infertility to male causes had a lower QOL score in the Mental Health domain. This was not observed among men in that study. Interestingly, we have found an distinct effect among men, and an impact in a different aspect of QOL (Physical versus Mental QOL) [19].

There are some limitations in the present study. The cross-sectional design does not allow inference of causality. It is possible to explore associations, but not to determine causal effects. Follow-up studies would be required to address this issue. Moreover, our study is based in a Brazilian sample, and cultural aspects may play a role in the identification of predictors and effects of depression and anxiety. However, it has been demonstrated that these symptoms have a relevant impact in several international samples. It is also important to observe that the tested models did not include other demographic, clinical and social variables, which could be potentially related to depression/anxiety and QOL, such as life stressors and social support. Finally, we point out that this is a clinically based investigation, which might interfere with the external validity of the findings.

## Conclusion

In this study, subthreshold levels of depression and anxiety were major predictors of quality of life. As such, it is crucial that health professionals involved with the treatment of infertile individuals should be particularly engaged in identifying even minor depressive and anxious symptomatology, and consequently providing these patients with adequate interventions, in order to keep their quality of life.

## Competing interests

The authors declare that they have no competing interests.

## Authors' contributions

JRC, DK and EPP participated in the design, coordination and conception of the study. EC performed the statistical analysis and conceived of the study. HE, MPF and FC took part in the theoretical discussions and helped to draft the manuscript. All authors read and approved the final manuscript.
